# Overlapping but Language-Specific Mechanisms in Morphosyntactic Processing in Highly Competent L2 Acquired at School Entry: fMRI Evidence From an Alternating Language Switching Task

**DOI:** 10.3389/fnhum.2021.728549

**Published:** 2021-11-26

**Authors:** Simin Meykadeh, Arsalan Golfam, Seyed Amir Hossein Batouli, Werner Sommer

**Affiliations:** ^1^Department of Linguistics, Tarbiat Modares University, Tehran, Iran; ^2^Institut für Psychologie, Humboldt-Universität zu Berlin, Berlin, Germany; ^3^Department of Neuroscience and Addiction Studies, School of Advanced Technologies in Medicine, Tehran University of Medical Sciences, Tehran, Iran; ^4^Department of Psychology, Zhejiang Normal University, Jinhua, China

**Keywords:** bilingualism, left pSTG, left pars opercularis, morphosyntax, switch cost asymmetry, Persian, Turkish, fMRI

## Abstract

Many bilingual individuals acquire their second language when entering primary school; however, very few studies have investigated morphosyntax processing in this population. Combining a whole-brain and region of interest (ROI)-based approach, we studied event-related fMRI during morphosyntactic processing, specifically person-number phi-features, in Turkish (L1) and Persian (L2) by highly proficient bilinguals who learned Persian at school entry. In a design with alternating language switching and pseudorandomized grammaticality conditions, two left-lateralized syntax-specific ROIs and 11 bilateral ROIs involved in executive functions (EF) were analyzed for the intensity of activation relative to a resting baseline. Our findings indicate a strong overlap of neural networks for L1 and L2, suggesting structural similarities of neuroanatomical organization. In all ROIs morphosyntactic processing invoked stronger activation in L1 than in L2. This may be a consequence of symmetrical switch costs in the alternating design used here, where the need for suppressing the non-required language is stronger for the dominant L1 when it is non-required as compared to the non-dominant L2, leading to a stronger rebound for L1 than L2 when the language is required. Both L1 and L2 revealed significant activation in syntax-specific areas in left hemisphere clusters and increased activation in EF-specific areas in right-hemisphere than left-hemisphere clusters, confirming syntax-specific functions of the left hemisphere, whereas the right hemisphere appears to subserve control functions required for switching languages. While previous reports indicate a leftward bias in planum temporale activation during auditory and linguistic processing, the present study shows the activation of the right planum temporale indicating its involvement in auditory attention. More pronounced grammaticality effect in left pars opercularis for L1 and in left pSTG for L2 indicate differences in the processing of morphosyntactic information in these brain regions. Nevertheless, the activation of pars opercularis and pSTG emphasize the centrality of these regions in the processing of person-number phi-features. Taken together, the present results confirm that morphosyntactic processing in bilinguals relates to composite, syntax-sensitive and EF-sensitive mechanisms in which some nodes of the language network are differentially involved.

## Introduction

Many studies on bilingualism focus either on very early vs. late exposure (age of acquisition, AoA) to the second language (L2), while many children from ethnic minorities around the world are being exposed to an L2 only at school entry as the language of instruction. Identification of the AoA cutoff is controversial and may not affect all language capacities equally. Arguably, modifications in the lexicon are least sensitive to AoA (Meisel et al., 2013), whereas automaticity and correctness of grammar processing appear to be at a disadvantage if L2 is acquired after age seven (e.g., Fabbro, 2001; Ullman, 2001b). Ullman (2001b) suggested that the AoA sensitivity of grammar involves greater reliance on declarative memory in L2 whereas for L1 procedural memory dominates. On this view, the storage and retrieval of words depend on distributed associative memory subserved by temporal-lobe circuits. In contrast, the acquisition and application of grammatical rules are subserved by frontal/basal-ganglia circuits. Therefore, lexicon and grammar are based on distinct computational components linked to different brain structures (Ullman, 2001a). Following the suggested cut-off period at age 7, the present study aims to shed more light on the effects of learning an L2 at a presumably critical age for grammatical rule acquisition. To this aim, we will compare morphosyntactic processing in L1 and L2 in highly proficient Turkish-Persian bilinguals.

Many neuroimaging studies have investigated syntactic processing in bilingual brains but there is little specific work on morphosyntactic parameters, such as inflection in a verbal agreement. Morphosyntax relates to the form-function association, that is, the relationship between the morphemes of a word and the sentence structure. As a notable exception, Wartenburger et al. (2003) recruited high-proficient very early and high- and low-proficient late German/Italian bilinguals and examined the influence of AoA and level of proficiency during a grammaticality judgment task, manipulating morphological markers. Their early bilinguals had been exposed to L2 since birth, while in late bilinguals mean AoA was 18.9 and 20.4 years, respectively, in the high- and low-proficiency groups. Both late bilingual groups showed more extensive activation of Broca’s region and subcortical structures in L2 while activation was identical for L1 and L2 in early acquisition bilinguals, suggesting that the neural basis for morphosyntactic processing depends on AoA even in late bilinguals with native-like L2 proficiency. The late bilingual’s higher activation was taken to reflect their greater difficulty of L2 processing due to AoA after puberty.

In their review, Cargnelutti et al. (2019) concluded that for L2 processing early bilinguals (AoA < age 6) engage a widespread network including the classical language areas, together with supporting cortical and subcortical regions related to general cognition, reflecting their constant efforts to manage both languages, especially L2, even when highly proficient. Similarly, Roncaglia-Denissen and Kotz (2016) suggested overlapping networks recruited during morphosyntactic processing in L1 and L2 and persistent AoA effects even at comparable proficiency levels. Consistently, neurosurgical language mapping studies in bilinguals indicate that the amount of neuroanatomical overlap between L1 and L2 decreases with AoA (Połczyńska and Bookheimer, 2020).

According to the neuroanatomical pathway model of Friederici et al. (2017), the processing of syntactic structures involves Broca’s area in the inferior frontal gyrus (IFG) of the left hemisphere and the superior temporal gyrus (STG) as major hubs. The posterior part of Broca’s area (pars opercularis, BA 44) appears to subserve strictly syntactic processing, whereas its anterior portion (pars triangularis, BA 45) is known to mainly support lexico-semantic processing. These two subregions of Broca’s area, BA 44 and BA 45, are connected to the temporal cortex by distinct dorsal and ventral fiber tracts, respectively. Given their target regions within the temporal cortex, the dorsal and ventral pathways appear to support syntactic vs. semantic processes, respectively. Sentence comprehension as a whole recruits a frontotemporal network that includes both Broca’s area and the posterior superior temporal gyrus (pSTG). Basically, in adult humans, there is a specific network including a functionally specified BA 44 that is structurally and functionally connected to the left pSTC. This neural circuit may thus be fundamental for the human syntactic capacity as the core of language (Friederici, 2017).

A fundamental factor in bilingual language competence is cognitive control or executive functions (EFs; Lerman and Obler, 2017). At least three core EFs are commonly distinguished, encompassing working memory, inhibition, and cognitive flexibility; these EFs likely represent distinct cognitive subsystems that nevertheless functionally overlap (Zink et al., 2021). Bilingualism has been argued to involve working memory resources for managing languages that constantly compete for selection (Antón et al., 2019).

According to the inhibitory control (IC) model (Green, 1998), an inhibitory mechanism is engaged in resolving conflicts between two simultaneously activated languages and inhibiting the non-target language to ensure the production of the target language. Therefore, after a switch from one language to the other, more effort is required to inhibit the residual activation of L1 than of L2, leading to larger switching costs from L1 to L2 than *vice versa*. However, the amount of inhibition required also depends on the speaker’s relative proficiency in each language, such that the dominant language must be inhibited to a larger extent than the weaker language (Ma et al., 2016). The literature revealed that bilinguals do not need to inhibit the nontarget language when they are highly proficient in two languages (for a review see Ma et al., 2016).

Based on cognitive and computational literature, Zink et al. (2021) recently argued that EFs are neither generated strictly top-down nor localized in specific brain areas; instead, they may be the emerging consequence of communication within a broad network of spatially and functionally dispersed brain systems that integrate different aspects of EFs. Therefore, we aimed to identify the neural correlates of EFs in our study using a whole-brain approach as a first step to properly take into account the distributed nature of information conveyed by BOLD signals.

A substantial number of neuroimaging studies have shown that shared regions are recruited for processing L1 and L2 (Abutalebi et al., 2001; Tan et al., 2003; Wartenburger et al., 2003; Perani and Abutalebi, 2005; Liu et al., 2010). However, using multivariate pattern analysis (MVPA), Xu et al. (2017) recently challenged the traditional single cortical mechanism hypothesis, demonstrating instead that L1 and L2 of Chinese-English bilinguals elicit brain activation in common regions but with notably distinguishable patterns. The authors suggested functional independence of neural computations underlying the representations of different languages in bilinguals.

The present study investigated Turkish (L1)/Persian (L2) bilinguals in terms of morphosyntactic processing. Turkish and Persian belong to the Altaic and Indo-Iranian subdivisions of the Indo-European language family, respectively, but share unmarked subject-object-verb (SOV) word orders (Comrie, 2009) and certain syntactic features, such as verbal agreements. Subject-verb agreement in Turkish and Persian entails the analysis of two phi-features, namely person and number. Thus, in both languages, verbs obligatorily agree in person and number with animate subjects, they have six grammatical persons and are inflected for three singular and three plural persons. According to the unified competition model (UCM; MacWhinney, 2005), the mechanisms of L1 learning are seen as a subset of the mechanisms of L2 learning. In particular, whenever a surface structure, such as morphosyntax, is shared, the mechanisms used in L1 will be transferred to process L2 (Roncaglia-Denissen and Kotz, 2016).

The objective of this study is to examine how a bilingual brain with L2-AoA at school entry is able to manage morphosyntactic information in L1 and L2. According to the model of Friederici et al. (2017), we predicted the involvement of a left-lateralized fronto-temporal network in regulating morphosyntax in our highly proficient young adult bilinguals. Furthermore, we were interested in the network related to EFs, required in managing L1 and L2 activation. Given that no neuroimaging study to date has examined the pattern of brain activity within the same individuals during morphosyntactic processing using a rapid language-switching paradigm, we aimed to contribute to the literature about morphosyntactic analysis of L1 and L2 in two SOV languages.

## Methods

### Participants

Participants were recruited among university students in Tehran who had Turkish-speaking parents, had spent most of their life in Turkish-speaking provinces of Iran, and had learned Persian at school from the age of 7. They reported to speak Turkish at home and with their families but had received their formal education in the Persian language, had spent at least 5 years (range 5–7) in a Persian-speaking city, and spoke both Turkish and Persian in their daily life. Initially, 41 healthy right-handed (Oldfield, 1971) adults who reported normal hearing participated. Five data sets were excluded because of aliasing artifacts and excessive movements. The final sample consisted of 36 participants (21 female, 15 male; mean age = 27.4, range = 22–34 years; mean years of education = 19.5 years). There were no significant sex differences in age or education.

Due to the lack of standardized proficiency tests for Turkish and Persian, the proficiency level was assessed by language-learning history (living in a Persian environment for at least 5 years), an interview conducted in Turkish and Persian, and self-ratings on 6-point Likert scales. Mean proficiency self-ratings in Persian were high with little difference between comprehension and production (6 vs. 5). All participants were similar in socioeconomic status as indexed by parent education and occupation (measured by the Hollingshead Four Factor Index of Social Status; Hollingshead, 1975). Hence, all participants were judged to have a high level of proficiency in both languages. Participants provided written informed consent and were reimbursed. The study was conducted according to the Helsinki regulations and approved by the Research Ethical Committee of Iran University of Medical Sciences (IR.IUMS.REC.1398.465).

### Materials

The material consisted of 64 Persian and 64 Turkish sentences, following the structure: Subject + Object + Verb. Verbs were regular and highly frequent, chosen from Anvari and Givi (2006) and Ketrez (2012), respectively. Only past tense transitive verbs of a similar kind of transitivity (direct) and without copula were used. Half of the sentences in each language were morphosyntactically correct, whereas the other half included person-number phi-feature agreement violations in the verb. In the correct conditions, a 1st and 3rd person singular subject was followed by a 1st and 3rd person singular verb, and a 1st and 3rd person plural subject was followed by a 1st and 3rd person plural verb, respectively. In contrast, in the Number and Person violation conditions, a 1st and 3rd person singular subject was followed by a 1st and 3rd person plural verb, and a 1st and 3rd person plural subject was followed by a 1st and 3rd person singular verb respectively as illustrated in Table 1.

**Table 1:** Examples for sentence materials.

Example in Turkish and Persian with transliterations and literal translations in parenthesis*	
**L1**	**L2**
*Number Violation*	
fnhum-15-728549-i0001.tif	fnhum-15-728549-i0002.tif
*Biz kitâe-lar-i-mizi gatirdim. We book-PL-HI-OBJ-CLT.Def bring-PAST-1SG (We brought our books).	*Ma_1.pl_ bedehkari-ye-mân râ pardâxtam_1.sg._ We debt-HI-OBJ-CLT.Def pay.PAST-1SG(We paid our debts.)
*Person Violation.*	
fnhum-15-728549-i0003.tif	fnhum-15-728549-i0004.tif
*Biz ev-ler-i-mizi sildular. We house-PL-HI-OBJ-CLT.Def clean-PAST-3PL (We cleaned our houses)	*Ma_1.pl_ nâme-ha-ye-tân râ ferestâdand_3.pl._ We letter-PL-HI-OBJ-CLT.Def send.PAST-3PL (We sent their letters.)
*Correct Agreement.*	
fnhum-15-728549-i0005.tif	fnhum-15-728549-i0006.tif
Man_1.sg_ pâltâr-e-mi yudum_1.sg._ I cloth-HI-OBJ-CLT.Def wash.PAST-1SG (I washed my cloth.)	Man_1.sg_ nazar-aš râ paziroftam_1.sg._ I offer-OBJ-CLT.Def accept.PAST-1SG (I accepted her/his offer.)

*The critical syllable is underlined. 1, First person; SG, Singular; Def, Definitive; PAST, Past; 3, Third person; PL, Plural; HI, Hiatus; OBJ-CLT, Objective clitic.

The critical syllable always occurred at the sentence-final position. All sentences are semantically correct. Syntactically correct and incorrect sentences were not derived from one another; hence, each sentence was presented only once whether correct or incorrect. The sentences were spoken by a female in natural tempo and prosody and stored in WAV format (sampling: 16-bit, 44 kHz).

### Procedure

The experiment consisted of a 2-h behavioral session and a 40-min fMRI session at a different time point. In the behavioral session, participants performed a Reading Span Test (Khodadadi et al., 2014) and were assessed for handedness and language proficiency. In the fMRI session participants were instructed about their task of judging grammatical but not semantic correctness of each sentence by pressing the button of a left (ungrammatical) or right (grammatical) response grip with the thumb. Sentences were presented *via* headphones using MATLAB’s Psychtoolbox.

The experiment used an event-related design, including four alternating rest and auditory sentence blocks and an alternating language switching paradigm. Each sentence block consisted of 32 runs and was preceded and followed by 30-s resting periods during which no stimuli were presented to provide hemodynamic baseline data (318 s total per block). Each run contained a 1-s beep sound, 3-s sentence presentation, and a response phase of 4-, 5-, or 6-s (*M* = 5 s). Due to the auditory nature of the task, a fixation cross was displayed throughout the experiment at the center of the screen. Within each switching block, the two languages continuously alternated (e.g., L1, L2, L1, L2 …). Additionally, grammatical and ungrammatical sentences of both languages were randomly intermixed within each block and presented in the same random order to all participants. The advantage of the alternating language switching paradigm over cued language switching, sequence-based language switching, or voluntary language switching, is to allow to prepare for the upcoming language switch, similar to preplanning during natural language processing (for more arguments, see Declerck and Philipp, 2015). Hence, based on the predictable language sequence, one can prepare for the upcoming language (but not for its grammaticality, which is random). In real-world, bilingual speakers are capable of using each of their languages appropriately and they can promptly switch from one language to the other. Here, we measured and compared switching costs from L1 to L2 and *vice versa*, in terms of RT and BOLD activity.

### Imaging

Functional T2^*^-weighted EPI-BOLD MRI data were obtained on a 3.0 Tesla Siemens Prisma MRI Scanner at National Brain Mapping Laboratory (NBML), using a sequential slice acquisition EPI sequence (TE: 30 ms, TR: 3,000 ms, flip angle: 90°, slice thickness: 3 mm, voxel size: 3 × 3 × 3 mm, matrix size: 64 × 64, FOV: 192 mm^2^, slice gap: 0 mm) with a 20-channel head coil and a functional scanning time of 1,290 s and 430 volumes. Each volume was composed of 45 axial slices. Structural images were acquired with a T1-weighted sequence, using a 3D inversion-recovery gradient-echo (MP-RAGE) sequence (TE: 3.53 ms, TR: 1,800 ms, flip angle: 7°, slice thickness: 1 mm, voxel size: 1 × 1 × 1 mm, matrix size: 256 × 256, FOV: 256 mm^2^, slice gap: 0 mm, duration: 5 min).

### Data Preprocessing

Images were analyzed using fMRI Expert Analysis Tool (FEAT) Version 6.00, part of FMRIB’s Software Library (FSL^1^), and based on previous works (Batouli and Saba, 2020; Batouli et al., 2020). Registration to high resolution structural and/or standard space images was carried out using FLIRT (Jenkinson and Smith, 2001 and Jenkinson et al., 2002). Functional datasets were preprocessed using the following analysis steps: motion correction with MCFLIRT (Jenkinson et al., 2002); slice-timing correction using Fourier-space time-series phase-shifting; non-brain removal with BET (Smith, 2002); spatial smoothing using a Gaussian kernel of FWHM 6.0 mm; multiplicative mean intensity normalization of the volume at each time point; and high pass temporal filtering (Gaussian-weighted least-squares straight line fitting, sigma = 50.0 s). In order to investigate the presence of artifacts or activation, exploratory ICA-based data analysis was conducted using MELODIC (Beckmann and Smith, 2004).

Statistical time-series analysis was carried out using FILM (FMRIB Improved Linear Model) with local autocorrelation correction (Woolrich et al., 2001) and a “z-score” was assigned to the corresponding BOLD signal. Higher-level analysis was carried out with FLAME (FMRIB’s Local Analysis of Mixed Effects; Beckmann et al., 2003; Woolrich et al., 2004; Woolrich, 2008). Next, cluster thresholding was implemented to reveal significantly activated clusters. Clusters with *z*-stat >2.3 and *p* < 0.05 were considered to be significantly activated. We repeated the analyses also with a stricter z-stat criterion (>3.1), in order to identify a stronger activation of the brain areas and, therefore, more robust results. Only trials (sentences) with correct responses were included in the analysis.

### ROI Analyses

In order to determine the effects of bilingualism on L1/L2 morphosyntactic processing, syntax-specific ROI analyses including left pars opercularis and left pSTG were performed in line with the model of Friederici et al. (2017). Additionally, to explore the EFs-specific network in our participants, ROI analyses were performed using a whole-brain approach. For each participant and each ROI, percent signal change (%SC) was computed as an intensity measure according to the Harvard-Oxford Atlas as implemented in FSL. The intensity was set as the dependent variable, while grammaticality, language, and hemisphere were independent variables. All statistical analyses were performed using SPSS v26 (IBM Corporation, Armonk, NY). The effects of interest for intensity at each ROI were analyzed with repeated measures analysis of variance (ANOVA) on factors language (L1 = Turkish, L2 = Persian), grammaticality (grammatical, ungrammatical), and hemisphere (left, right). To control for multiple comparisons, Bonferroni correction was applied and only significant results are reported. Critical alpha was set to 0.05/2 = 0.025 (omnibus ANOVAs) and 0.05/8 = 0.006 (*Post hoc* t-test), for syntax-specific network. Also, the alpha level was chosen at 05/13 = 0.0038 (omnibus ANOVAs), and 0.05/26 = 0.0019 (*Post hoc* t-test) for EFs-specific network.

## Results

### Behavioral Results

**Figure 1:**
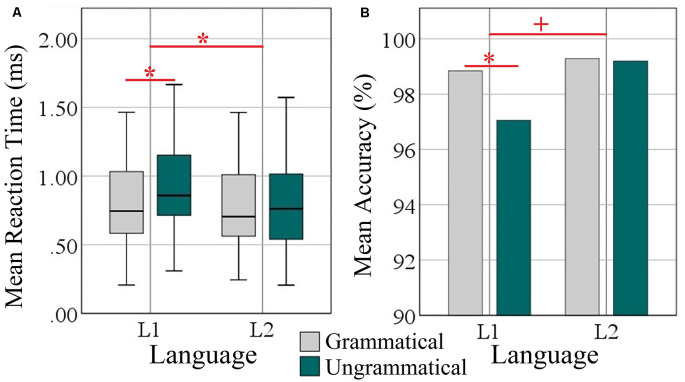
Behavioral analysis of L1 (Turkish) and L2 (Persian). **(A)** Box plots of mean reaction time over grammatical and ungrammatical conditions per language in milliseconds. **(B)** Bar plots of mean accuracy as a percentage of correct answers over grammatical and ungrammatical conditions per language. Significant effects are indicated by the horizontal red bar; statistical difference (ANOVA and *Post hoc* two-tailed t-test) *p < 0.05. The plus sign denotes trends at *p* < 0.05.

Performance results are depicted in Figure 1. On average, participants correctly classified the grammaticality of more than 99% of the sentences. Accuracy rates were very high and on average 98.96% (±1.18) for L1 and 99.61% (±0.79) for L2. Mean RTs to L1 and L2 sentences were 0.88 (±0.37) vs. 0.78 (±0.36) ms. The accuracy and RT data were submitted to 2 × 2 within-subject ANOVAs with factors grammaticality and language.

Regarding accuracy, there were significant main effects of grammaticality (*F*_(1, 34)_ = 4.73, *p* = 0.037, $\eta_{p}^{2}$ = 0.122) and language (*F*_(1, 34)_ = 8.98, *p* = 0.005, $\eta_{p}^{2}$ = 0.209). The interaction Grammaticality × Language was a strong trend (*F*_(1, 34)_ = 3.95, *p* = 0.054, $\eta_{p}^{2}$ = 0.104). *Post hoc* paired samples *t*-tests revealed a significant difference of the accuracy scores between the two conditions only in L1 (*t*_(35)_ = 2.66, *p* = 0.012) but not for L2 (*t*_(35)_ = 0.167, *p* = 0.869).

Regarding reaction times, ANOVA revealed main effects of Grammaticality (*F*_(1, 34)_ = 12.75, *p* = 0.001, $\eta_{p}^{2}$ = 0.273) and Language (*F*_(1, 34)_ = 18.55, *p* = 0.000, $\eta_{p}^{2}$ = 0.353) and a significant interaction of both factors (*F*_(1, 34)_ = 7.38, *p* = 0.010, $\eta_{p}^{2}$ = 0.179). *Post-hoc* t-tests showed that the difference between languages was specific to L1 (*t*_(35)_ = 4.51, *p* = 0.000) but not to L2 (*t*_(35)_ = 0.610, *p* = 0.546; *M* = 0.115 vs. 0.016 ms).

### Whole-Brain Activation Results

**Figure 2:**
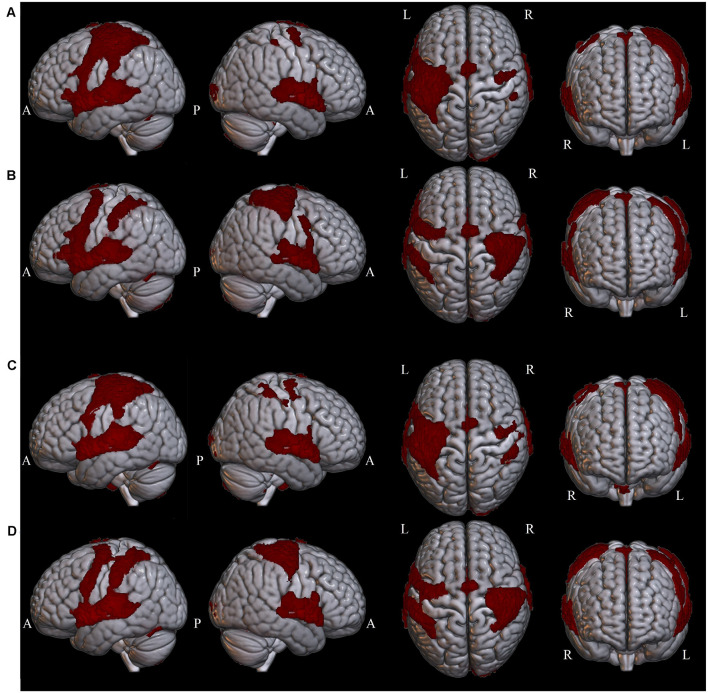
Whole-brain clusters are projected onto surface templates using MRIcroGL software in four conditions: **(A)** Grammatical L1, **(B)** Ungrammatical L1, **(C)** Grammatical L2, **(D)** Ungrammatical L2.

Widespread significant BOLD activation (Figure 2) was found during the presentation of the sentences of L1 (Table 2) and L2 (Table 3) in regions commonly associated with language. Relative to the baseline, there was dominant activation in the left pars opercularis and left pSTG, which are held to be responsible for the processing of pure syntactic information (Friederici et al., 2017). Furthermore, the right superior frontal gyrus (SFG), bilateral anterior cingulate gyrus (ACG), bilateral superior parietal lobule (SPL), bilateral supplementary motor area (SMA), bilateral temporal pole, bilateral precentral, bilateral postcentral, bilateral paracingulate, right Putamen, bilateral planum temporale (PT), and bilateral cerebellum were found to be activated for both L1 and L2. Thalamus and caudate were activated only for L1. In the following, we will focus on the two left-lateralized ROIs (pars opercularis and pSTG) and eleven bilateral ROIs (as described above), allowing for statistical evaluation of language and grammaticality effects in signal intensity.

**Table 2:** Brain regions involved in L1 separately for each condition compared to the baseline (whole-brain analyses).

Cerebral area	Coordinates			Cluster index	No. of voxels	*Z*-value
	*x*	*y*	*z*			
*Grammatical sentences*						
L Postcentral Gyrus	−46	−26	52	1	18,463	7.24
L Temporal Pole	−56	8	−8			7.09
L Thalamus	−14	−16	10			6.68
L STG, posterior	−63	−20	6			6.88
L IFG, pars opercularis	−54	13	4			5.81
R Cerebellum	22	−54	−20	2	10,577	8.05
L Cerebellum	−20	−64	−54			7.53
R STG, anterior	60	2	−2	3	3,919	6.82
R STG, posterior	48	−32	6			6.61
R Temporal Pole	54	14	−10			6.32
R Planum Temporale	62	−14	4			5.97
L SMA	−2	0	58	4	2,708	8.29
L Paracingulate Gyrus	−2	10	50			7.58
L ACG	8	14	38			5
R SFG	6	2	76			4.22
R Putamen	24	2	6	5	351	5.58
R Caudate	10	6	8			3.91
R Precentral Gyrus	36	−14	72	6	237	4.47
R Postcentral Gyrus	48	−26	48	7	172	4.04
R SPL	40	−34	48			3.99
*Ungrammatical sentences*						
L Temporal Pole	−58	6	−8	1	14,561	6.77
L STG, posterior	−66	−16	6			6.66
L IFG, pars opercularis	−50	10	4			6.64
L SPL	−46	−40	50			6.53
L Precentral Gyrus	−44	4	40			6.52
L Cerebellum	−14	−56	−16	2	11,225	8.55
R Cerebellum	26	−66	−54			7.5
R Putamen	26	0	6	3	5,022	6.49
R STG, anterior	62	4	−2			6.16
R Temporal Pole	54	14	−10			5.83
R STG, posterior	48	−32	4			5.65
R Thalamus	16	−16	6			5.57
R Postcentral Gyrus	46	−22	48	4	4,865	7.25
R Precentral Gyrus	40	−22	54			7.16
L Paracingulate Gyrus	−6	12	40	5	3,009	6.99
L SMA	−2	2	56			7.5
L ACG	8	14	38			6.99
R SFG	8	0	78			4.6

**Table 3:** Brain regions involved in L2 separately for each condition compared to the baseline (whole-brain analyses).

Cerebral area	Coordinates			Cluster index	No. of voxels	*Z*
	*x*	*y*	*z*			
*Grammatical sentences*						
L Postcentral Gyrus	−46	−26	52	1	15,938	7.48
L SPL	−40	−38	48			7.24
L Planum Temporale	−64	−22	10			7.05
L STG, posterior	−66	−18	8			7.14
L IFG, pars opercularis	−52	8	4			5.71
R Cerebellum	24	−54	−22	2	10,660	7.94
L Cerebellum	−20	−64	−54			7.48
R STG, anterior	62	2	0	3	3,909	6.92
R STG, posterior	48	−34	4			6.64
R Temporal Pole	52	14	−8			6.57
R Planum Temporale	62	−14	4			6.1
L SMA	−2	0	58	4	2,342	7.61
R ACG	8	14	38			5.04
R SFG	12	−2	76			3.79
L paracingulate	−3	8	50	5	1,297	4.35
R Postcentral Gyrus	48	−32	56			4.82
R SPL	34	−50	44	6	249	4.15
R Precentral Gyrus	36	−12	68	7	165	4.5
R Putamen	24	2	6			5.62
*Ungrammatical sentences*						
L Planum Temporale	−58	−10	6	1	11,900	6.68
L STG, posterior	−68	−16	8			6.65
L Temporal Pole	−58	8	−8			6.64
L SPL	−46	−40	50			6.3
L IFG, pars opercularis	−50	8	2			6.24
L Cerebellum	−14	−54	−16	2	9,432	7.72
R Cerebellum	12	−68	−52			6.79
R Precentral Gyrus	44	−14	60	3	4,328	7.08
R Postcentral Gyrus	46	−24	48			7.07
R Putamen	26	−2	6	4	4,273	7.09
R STG, anterior	62	4	−2			6.15
R Temporal Pole	52	16	−10			5.67
R Putamen	28	0	−6			5.54
R Postcentral Gyrus	66	−6	8			5.5
R STG, posterior	46	−30	6			5.42
R SFG	6	−3	70			4.32
R ACG	10	14	40			3.81
L SMA	−2	4	52	5	2,663	7.34
L ACG	−4	10	44			6.94
R Paracingulate Gyrus	6	8	48			6.57

### Syntax-Specific ROIs

**Figure 3:**
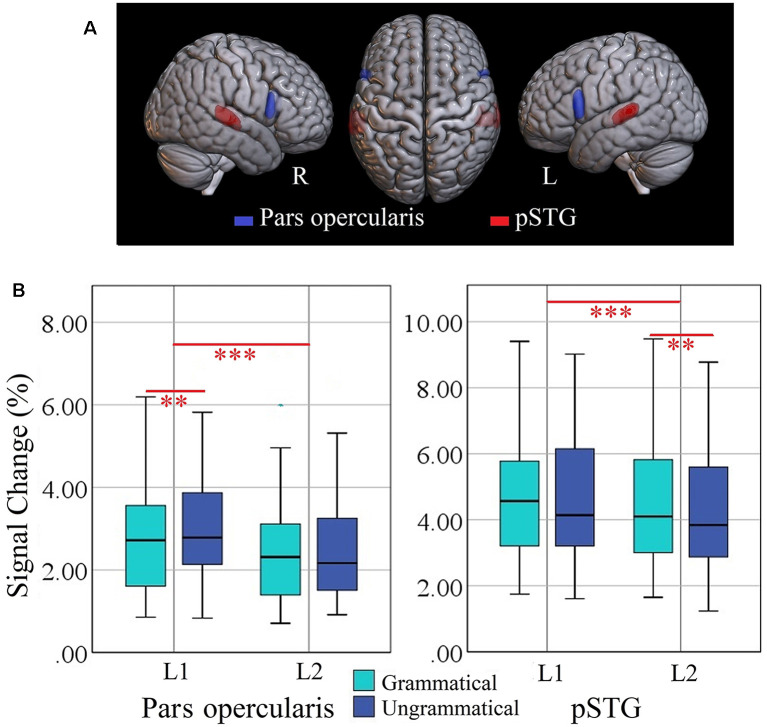
**(A)** Locations of ROIs representing pars opercularis (blue) and posterior superior temporal gyrus (pSTG, red). **(B)** Box plots of percent signal change (%SC) values, for each language (L1 = Turkish; L2 = Persian) and condition per ROI in the left hemisphere. Significant effects are indicated by the horizontal red bar (repeated measures ANOVA, Bonferroni-corrected ***p < 0.025; two-tailed t-test, Bonferroni-corrected **p < 0.006). ROI, region of interest.

The locations of the two ROIs are rendered in Figure 3, together with boxplots of signal intensity (%SC) relative to baseline. For the left pars opercularis, the main effect of language (*F*_(1, 34)_ = 18.0, *p* = 0.000, $\eta_{p}^{2}$ = 0.132) and the interaction of Grammaticality × Language (*F*_(1, 34)_ = 8.9, *p* = 0.006, $\eta_{p}^{2}$ = 0.205) were significant. Also, there was a trend for a main effect of grammaticality (*F*_(1, 34)_ = 5.2, *p* = 0.029, $\eta_{p}^{2}$ = 0.219 ; Bonferroni-adjusted alpha level = 0.025). *Post hoc* analyses of the interaction revealed a significant grammaticality effect for L1 (*t*_(34)_ = 3.09, *p* = 0.004) but not for L2 (*t*_(34)_ = −0.49, *p* = 0.622; *M* = 0.33 vs. −0.03%SC; critical alpha = 0.006).

The ANOVA for the left pSTG revealed significant main effects of language (*F*_(1, 34)_ = 14.1, *p* = 0.001, $\eta_{p}^{2}$ = 0.294) and grammaticality (*F*_(1, 34)_ = 9.5, *p* = 0.004, $\eta_{p}^{2}$ = 0.219). The interaction Language × Grammaticality was not significant (*F*_(1, 34)_ = 0.390, *p* = 0.538, $\eta_{p}^{2}$ = 0.011). Separate *t*-test of the grammaticality effects for each language showed that it was significant for L2 (*t*_(34)_ = −3.02, *p* = 0.0047) but not for L1 (*t*_(34)_ = −1.80, *p* = 0.080; *M* = −0.33 vs. −0.23%SC). The grammaticality effects of syntax-specific ROIs are presented in Table 4.

**Table 4:** Grammaticality effect of left-lateralized syntax-specific ROIs in separate languages.

Region	Language	Mean PSC (SD)	*t*-value
Pars opercularis	L1	0.332 (0.63)	3.099**
	L2	−0.034 (0.40)	−0.498
pSTG	L1	−0.237 (0.77)	−1.803
	L2	−0.335 (0.65)	−3.021**

**P < 0.006 (2-tailed *t*-test, Bonferroni-corrected). ROI, region of interest.

### Data-Driven EFs-Specific ROIs

The 2 × 2 × 2 ANOVAs of the ROIs related to cognitive control yielded significant interactions of Grammaticality × Hemisphere for ACG (*F*_(1, 34)_ = 19.48, *p* = 0.0001, $\eta_{p}^{2}$ = 0.364), SFG (*F*_(1, 34)_ = 15.91, *p* = 0.0003, $\eta_{p}^{2}$ = 0.319), SPL (*F*_(1, 34)_ = 43.42, *p* = 0.0000, $\eta_{p}^{2}$ = 0.561) and precentral gyrus (*F*_(1, 34)_ = 16.56, *p* = 0.0002, $\eta_{p}^{2}$ = 0.328), significant interactions of Language × Hemisphere for the planum temporale (*F*_(1, 34)_ = 17.29, *p* = 0.0002, $\eta_{p}^{2}$ = 0.337), significant main effects of Language for ACG (*F*_(1, 34)_ = 18.1, *p* = 0.0001, $\eta_{p}^{2}$ = 0.347), SMA (*F*_(1, 34)_ = 19.71, *p* = 0.0000, $\eta_{p}^{2}$ = 0.367), and paracingulate gyrus (*F*_(1, 34)_ = 34.12, *p* = 0.0000, $\eta_{p}^{2}$ = 0.501), a significant main effect of Grammaticality for the paracingulate gyrus (*F*_(1, 34)_ = 11.1, *p* = 0.002, $\eta_{p}^{2}$ = 0.247) and planum temporale (*F*_(1, 34)_ = 56.76, *p* = 0.000, $\eta_{p}^{2}$ = 0.625) and a significant main effect of Hemisphere for the planum temporale (*F*_(1, 34)_ = 23.83, *p* = 0.000, $\eta_{p}^{2}$ = 0.412). Also, the interaction of Grammaticality × Hemisphere was a trend (*F*_(1, 34)_ = 8.61, *p* = 0.0059, $\eta_{p}^{2}$ = 0.202; Bonferroni-adjusted alpha = 0.0038), suggesting that the omnibus Grammaticality effect was partly driven by a hemispheric difference. There were no significant effects in the other four regions (cerebellum, postcentral, putamen and temporal pole; *F*s < 1).

**Figure 4:**
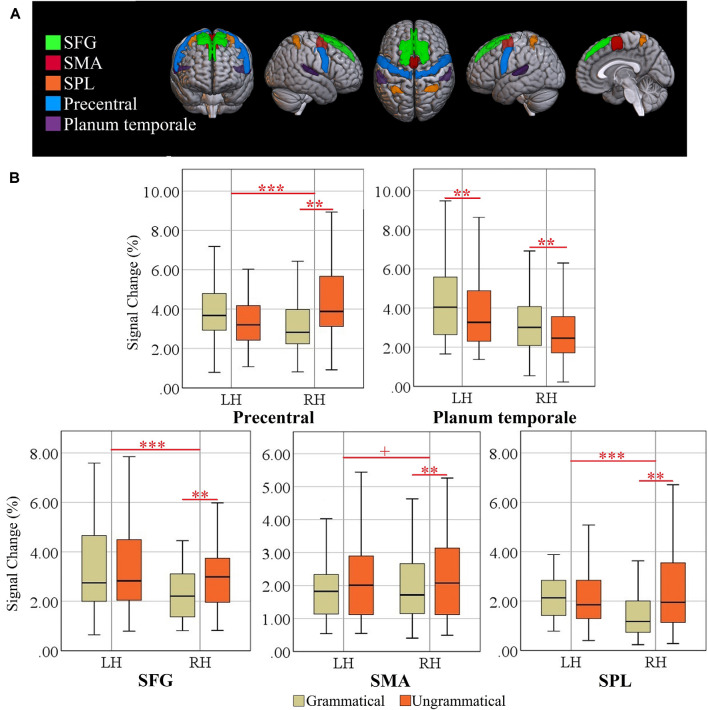
**(A)** Locations of ROIs. Superior frontal gyrus (SFG), supplementary motor area (SMA), superior parietal lobule (SPL), precentral gyrus, and planum temporale (PT). **(B)** Box plots of percent signal change (%SC) for left and right hemisphere (LH vs. RH) and grammatical and ungrammatical conditions. Significant effects are indicated by the horizontal red bar (repeated measures ANOVA, ***p < 0.0041; *Post-hoc* ANOVA, **p < 0.0020, Bonferroni-corrected). The plus sign denotes trends at *p* < 0.0041 (Bonferroni-corrected).

A *post hoc* repeated measure ANOVA of factors grammaticality and language for each hemisphere confirmed that the grammaticality effect was significant only in the right SFG (*F*_(1, 34)_ = 24.56, *p* = 0.0000, $\eta_{p}^{2}$ = 0.419), right SPL (*F*_(1, 34)_ = 20.58, *p* = 0.0000, $\eta_{p}^{2}$ = 0.377), right SMA (*F*_(1, 34)_ = 14.69, *p* = 0.0005, $\eta_{p}^{2}$ = 0.302) and right precentral (*F*_(1, 34)_ = 38.97, *p* = 0.0000, $\eta_{p}^{2}$ = 0.534) but not in the left regions (*F* < 1; Critical alpha = 0.0019). Moreover, there were significant grammaticality effects for the planum temporale in both right (*F*_(1, 34)_ = 44.55, *p* = 0.0000, $\eta_{p}^{2}$ = 0.567) and left (*F*_(1, 34)_ = 27.01, *p* = 0.0000, $\eta_{p}^{2}$ = 0.443) hemisphere. When multiple testing was taken into account using Bonferroni adjustment, no grammaticality effect in the right ACG was observed. The locations of the five significant ROIs for grammaticality are rendered in Figure 4, together with box plots of signal intensity (%SC) relative to baseline. The grammaticality effects of EFs-specific ROIs are presented in Table 5.

**Table 5:** Grammaticality effect of EFs-specific ROIs per hemisphere.

**Region**	Left hemisphere		Right hemisphere	
	Mean PSC (SD)	*t*-value	Mean PSC (SD)	*t*-value
SFG	−0.096 (1.11)	−0.726	0.603 (0.96)	5.256***
SMA	0.101 (0.87)	0.969	0.335 (0.66)	4.213***
SPL	−0.158 (0.87)	−1.519	0.865 (1.22)	5.908***
Precentral	−0.677 (2.32)	−2.437	1.061 (1.16)	7.619***
PT	−0.400 (0.58)	−5.714***	−0.417 (0.45)	−7.652***

***P < 0.0019 (2-tailed t-test, Bonferroni-corrected). EFs, executive functions.

## Discussion

The present study compared the neural mechanisms underlying morphosyntactic processing of L1 and L2 acquired from school entry and achieving high competence. The sentence materials randomly varied morphosyntactic correctness and alternated between L1 and L2, requiring alternating engagement and disengagement between languages (Green and Abutalebi, 2013). Paradis’ activation threshold hypothesis (Paradis, 1993, 2001) suggests that the retrieval of lexical items in a multilingual lexicon requires a minimum amount of activation while competing alternatives are inhibited (Hervais-Adelman et al., 2011). In bilinguals, the amount of inhibition depends on the relative language dominance (Green, 1998). Hence, we targeted both syntax-sensitive and EFs-sensitive ROIs.

In all ROIs, L1 invoked a greater activation than L2, which is opposite to the findings of the only other specific work on morphosyntax (Wartenburger et al., 2003). Although these two studies differed in many other respects, such as stimulus modality, word order patterns of languages used (in both Italian and German, argument and its verb occur in succession), we suggest that the most important difference accounting for the contrasting results is the frequent and regular switching of languages in the present design. The activation threshold hypothesis (Paradis, 1993, 2001) argues that during code-switching or language-mixing, bilinguals adopt one language as the base or matrix language and bring in the other language when required as a “guest” language. In consequence, both L1 and L2 are active but the base language is more strongly activated (Green, 1998). According to Zhu et al. (2020), the higher activation of the base language (presumably L1) leads to asymmetrical switch effects. The strong suppression of L1 during L2 sentence processing has to be overcome when there is a switch back to L1 input, resulting in higher activation in the ROIs involved in suppression but also impaired performance of L1. This account of the fMRI results is also in line with our performance results, with lower RT switch cost for L2 than L1, that is, switching into the nondominant language (L2). In contrast, for switching into the dominant language (L1) it takes longer to overcome the prior inhibition applied on this language. In other words, because L2 is the weaker language, increased cognitive control is required to re-activate L2 after L1 production (Zhu et al., 2020). Hence, our participants may have relied more on their L1 than L2.

Importantly, all ROIs were activated for both L1 and L2 (please see box plots in Figures 3, 4), suggesting convergent neuroanatomical networks and demonstrating the benefits of typologically shared linguistic surface structures. This is in line with the unified competition model (MacWhinney, 2005) viewing the mechanisms of L1 learning as a subset of the mechanisms of L2 learning. In contrast, the consistent activation of all ROIs across both languages contradicts the declarative/procedural model (Ullman, 2001a, b), postulating a greater reliance on declarative memory in L2. Our results strongly suggest the engagement of the same neural structures responsible for morphosyntactic processing in both L1 and L2.

We found consistent sensitivity of our setup for morphosyntactic processing in two syntax-specific ROIs, consisting in higher activity for ungrammatical than grammatical sentences. In addition to supporting the functional segregation within BA 44 and BA 45 (Makuuchi et al., 2009; Obleser et al., 2011; Goucha and Friederici, 2015), the coordination of activity in pSTG and left Broca’s area through a dorsal pathway (Friederici et al., 2017) was confirmed in the present study, suggesting the fundamental role of pars opercularis and pSTG in representing the core computational faculty of human language. Critically, this activation pattern enables us to associate the processing of person-number phi-features to specific neuroanatomical regions and to outline a map of phi-features in the brain. Furthermore, an interesting asymmetry was found in the grammaticality effect (Table 4) in L1 and L2. The left pars opercularis showed stronger activation for ungrammatical sentences in L1; in contrast, left STG showed higher activation in ungrammatical than grammatical sentences in L2. Our finding of different activation patterns in the same regions seem to be consistent with the findings of a recent study by Xu et al. (2017). Even in brain regions showing similar activations for the two languages, we observed that morphosyntax is differentially represented in native and nonnative languages. Taken together, we show an overlapping pattern of activation between L1 and L2 but also a partial specialization for L1 in left pars opercularis and for L2 in left pSTG.

Given the greater reliance of our participants on L1, we assume that there is a relationship between the degree of overlap of language regions for L1 and L2 and AoA. Very recently, Połczyńska and Bookheimer (2020) reviewed neurosurgical language mapping studies with different cutoff ages (i.e., 5, 6, 7, and later), concluding that earlier acquired L2 appeared to have more neuroanatomic overlap of representations for L1, whereas later acquired L2 showed more divergence. Further, as reported by Fabbro (2001), the representation of grammatical aspects of languages seem to differ between L1 and L2 if L2 is acquired after the age of 7, with less automatic processing and more errors than for the native language. Hence, we would expect that in the present design also, the overlap would increase with lower AoA and decrease with later AoA of L2.

Among the 11 EFs-specific ROIs, a network of predominantly right-lateralized cortical regions including the SPL, SMA, SFG, and precentral gyrus as well as the bilateral planum temporale showed effects of grammaticality, strongly suggesting that these brain regions are additionally involved in language switching, in line with current neural models of bilingual control (Luk et al., 2011; Green and Abutalebi, 2013) and findings from neuroimaging research, suggesting the involvement of these brain areas in language control (Rodriguez-Fornells et al., 2002; Wang et al., 2007, 2009; Abutalebi et al., 2008; Hernandez, 2009; Hervais-Adelman et al., 2011). Although a leftward bias in planum temporale asymmetry in auditory and linguistic processing has been previously reported, Hirnstein et al. (2013) suggested that the right planum temporale is not only critical for stimulus selection in dichotic listening but, beyond this function, is also involved in (stimulus-driven), auditory attention. Taken together, we propose that the fronto-parietal network described above is fundamentally responsible for general control processes invoked by language switching and auditory processing.

In conclusion, the present data on morphosyntactic processing in individuals who acquired their L2 at school entry indicate very similar processing in both L1 and L2. Possibly, due to the required language switching, the processing of L1 was more effortful than for L2. The dissociation of grammaticality effects in pars opercularis and pSTG to L1 and L2, as a further important result of our study, implying differences in the processing of morphosyntactic information for L1 and L2 in these brain areas.

## Data Availability Statement

The datasets presented in this article are not readily available because participants did not provide permission for data to be shared publicly. Requests to access the datasets should be directed to SM, a.meykadeh@modares.ac.ir.

## Ethics Statement

The studies involving human participants were reviewed and approved by Research Ethical Committee of Iran University of Medical Sciences (IR.IUMS.REC.1398.465). The patients/participants provided their written informed consent to participate in this study.

## Author Contributions

SM planned the study, collected and analyzed the data, and wrote the manuscript. AG supervised the work and provided valuable laboratory resources. SB contributed to the planning of the work and supervised data analysis. WS contributed to the planning of the work, supervised data analysis, and edited the manuscript. All authors contributed to the article and approved the submitted version.

## Conflict of Interest

The authors declare that the research was conducted in the absence of any commercial or financial relationships that could be construed as a potential conflict of interest.

## Publisher’s Note

All claims expressed in this article are solely those of the authors and do not necessarily represent those of their affiliated organizations, or those of the publisher, the editors and the reviewers. Any product that may be evaluated in this article, or claim that may be made by its manufacturer, is not guaranteed or endorsed by the publisher.

## Footnotes

^1^ www.fmrib.ox.ac.uk/fsl
